# Itchy Capillary Malformations: Unusual Appearance of Meyerson Phenomenon, a Case Series

**DOI:** 10.3390/pediatric13010019

**Published:** 2021-03-16

**Authors:** Manuel Sanchez-Diaz, Trinidad Montero-Vilchez, Luis Salvador-Rodriguez, Alejandro Molina-Leyva, Salvador Arias-Santiago, Jesús Tercedor-Sanchez

**Affiliations:** 1 Dermatology Unit, IBS Granada, Hospital Universitario Virgen de las Nieves, 18012 Granada, Spain; manolo.94.sanchez@gmail.com (M.S.-D.); tmonterov@gmail.com (T.M.-V.); l.salvador.rodriguez1991@gmail.com (L.S.-R.); alejandromolinaleyva@gmail.com (A.M.-L.); jesustercedor@gmail.com (J.T.-S.); 2 Granada School of Medicine, Granada University, 18016 Granada, Spain; 3 Pediatric Dermatology Unit, IBS Granada, Hospital Universitario Virgen de las Nieves, 18012 Granada, Spain

**Keywords:** eczema, vascular malformation, capillary malformation

## Abstract

Meyerson phenomenon, also known as “halo-eczema,” has been widely described over melanocytic and non-melanocytic lesions. However, its appearance over vascular anomalies is rarely observed and could lead to diagnostic errors. A case study of five patients aged between four months and two years is reported. These patients developed unique erythematous and pruritic scaly patches, being diagnosed and treated as fungal infections. Due to the lack of response to the treatment, they were referred to the pediatric dermatology practice, where the diagnosis of Meyerson phenomenon over capillary malformations was made. Topical treatment with corticosteroids led to improvement in all cases. Although Meyerson phenomenon developing over vascular anomalies is a rare condition, it is important for pediatricians and dermatologists to assess it as a part of the differential diagnosis when treating a patient with skin lesions. Recognizing this phenomenon will prevent diagnostic and therapeutic errors.

## 1. Introduction

Meyerson in 1971 first described two patients who presented with erythema, pruritus and desquamation over pre-existing melanocytic nevi and whose lesions improved after treatment with topical corticosteroids [1]. Since then, this phenomenon has been known as “Meyerson Phenomenon” or “Halo-Eczema” and has been described in a variety of pigmented and non-pigmented lesions. Most of the cases reported in children have been associated with congenital and acquired melanocytic nevi [2,3,4,5]. In adult patients, this phenomenon has been also described in nevi and melanoma [6]. However, only a few cases over vascular anomalies have been reported [7,8,9,10]. As the appearance of this phenomenon over vascular anomalies is rarely observed in pediatrics and general dermatology consultations, initial diagnostic errors are common. Therefore, recognizing its clinical characteristics is important for pediatricians and dermatologists to prevent diagnostic and therapeutic mistakes.

## 2. Case Presentation

A case series of five patients who presented with pruritic erythematous lesions which developed over pre-existing cutaneous vascular anomalies is reported. Patients were three males and two females, aged between four months and three years. All of them were healthy and had no relevant medical history, including the lack of criteria for atopic dermatitis. Patients had been diagnosed as having capillary malformations on different skin locations. In all the cases, the skin lesions were located over these vascular anomalies. The overview of patient’s characteristics and the location of the capillary malformations can be seen in Table 1. All the patients were tested for the diagnosis of fungal infection with fungal cultures, which were eventually negative. Because of the lack of diagnosis, they were referred to the pediatric dermatology unit.

**Figure 1:**
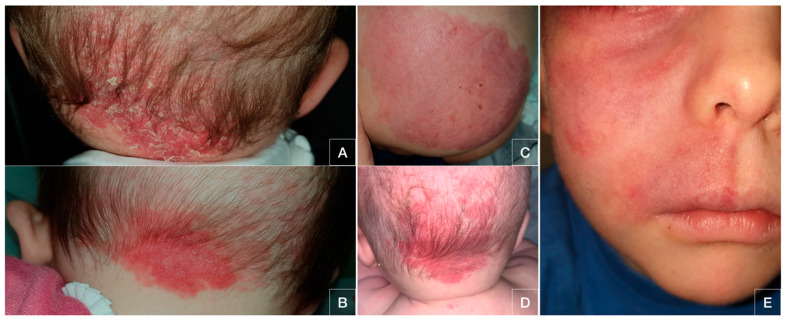
Eczematous patches corresponding to Meyerson phenomenon over capillary malformation. Patients: 1 (**A**), 2 (**B**), 3 (**C**), 4 (**D**) and 5 (**E**).

Patients did not identify any association with triggering events nor application of topical products. Only in the case of patient number 5 did the lesions develop after a laser treatment. Physical examination showed poorly defined erythematous-scaling patches in all cases. These lesions were located on the cheek, right hemifacial skin, nape of the neck and gluteal skin (see Figure 1).

Due to these clinical characteristics and their appearance over pre-existing cutaneous anomalies, the diagnosis of Meyerson phenomenon over vascular anomalies was made. Low-potency hydrocortisone-based topical corticosteroids were prescribed once a day for one week. Complete resolution of the condition was achieved in four cases. Only patient number 3 presented with recurrent eczema when the treatment was finished. In this case, a maintenance treatment with topical corticosteroids twice a week for four weeks was started, and complete improvement was finally achieved.

## 3. Discussion

Meyerson phenomenon or “halo-eczema” developing over vascular anomalies is rarely recognized in pediatric and dermatologic consultations, and there are few reports concerning this issue [7,8,9,10]. It consists of pruritic, erythematous and scaling patches developing over pre-existing cutaneous lesions. Although the etiology is unknown, there are multiple pathogenic theories to explain the appearance of this phenomenon in vascular anomalies. On the one hand, as the exact mechanism regarding how this phenomenon is associated with the underlying disease is still not clear, its potential actual influence on the common vascular anomalies classifications, such as the International Society for the Study of Vascular Anomalies (ISSVA), remains to be established. On the other hand, the appearance of eczema coexisting with a skin lesion without pathogenic relationship to the lesion itself could be misdiagnosed as Meyerson phenomenon.

**Figure 2:**
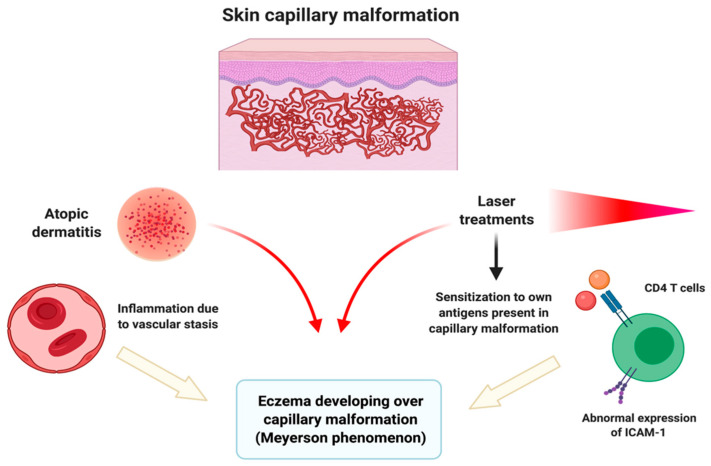
Pathogenic pathways probably involved in the development of Meyerson phenomenon over capillary malformations. Atopic dermatitis itself might be responsible for the appearance of eczemas over any skin lesion. Vascular stasis and sensitization to own antigens present in capillary malformations might also trigger an inflammatory eczematous reaction. CD4+ T cells and abnormal expression of ICAM-1 have also been involved in its pathogenesis. Laser treatments are related to both the improvement and the development of Meyerson phenomenon. Created with Biorender.

Atopic dermatitis itself might be responsible for the development of eczemas over any skin lesion; vascular stasis and sensitization to own antigens present in capillary malformations might also trigger an inflammatory eczematous reaction [9]. As we report in the case of the patient number 5, laser treatment might be a triggering event of Meyerson phenomenon. This fact might be erroneously considered by parents as a poor response to laser treatment. However, it has also been reported as a possible treatment which might improve the eczematous lesions over capillary malformations [8]. An overview of possible pathogenic pathways can be seen in Figure 2.

When assessing a patient, pediatricians and dermatologists should recognize that the appearance of pruritic eczematous patches over any type of pre-existing cutaneous lesion is very suggestive of Meyerson phenomenon. These clinical characteristics and the good response to topical corticosteroid treatment are sufficient to make the diagnosis, so skin biopsies are not routinely necessary. In case of diagnostic doubt, a skin biopsy can be performed. Histopathology usually shows acanthosis with marked spongiosis in the epidermis and perivascular lymphocytic infiltration in the dermis [7], which are features usually observed in eczematous disorders.

Although spontaneous resolution of Meyerson phenomenon is possible, symptomatic treatment is usually chosen. It is based on topical low-potency corticosteroids or calcineurin inhibitors, improving in most cases. The good response to topical treatment is a typical characteristic which supports the clinical diagnosis.

## 4. Conclusions

In conclusion, although Meyerson phenomenon over vascular anomalies is a rare condition, it is important for pediatricians and dermatologists to assess it as a part of the differential diagnosis when treating a patient with skin disorders. Pruritus, appearance over pre-existing lesions, erythematous-scaling patches and good response to topical corticosteroids are clinical keys for the diagnosis. Recognizing this phenomenon will prevent diagnostic and therapeutic mistakes.

## Figures and Tables

**Table 1:** Overview of patient’s characteristics.

Patient	Gender	Age	Fungal Cultures	Location of Capillary Malformations	Evolution (Treated with Topical Hydrocortisone)
1	Male	6 months	Negative	Nape of the neck	Good response
2	Female	7 months		Nape of the neck	Good response
3	Male	11 months		Gluteal skin	Recurrent eczema
4	Female	4 months		Nape of the neck	Good response
5	Male	2 years		Right hemifacial skin	Good response (appeared after laser treatment)

## Data Availability

No new data were created or analyzed in this study. Data sharing is not applicable to this article.
